# The Microbiome of an Invasive Antarctic insect, *Eretmoptera Murphyi* (Diptera: Chironomidae), and its Potential Role in Nutrient Cycling

**DOI:** 10.1007/s00248-026-02706-5

**Published:** 2026-02-28

**Authors:** Octavia D. M. Brayley, Kirsty McCready, Shengwei Liu, Peter Convey, Yin Chen, Sami Ullah, Nicholas Teets, Scott A.L. Hayward

**Affiliations:** 1 School of Biosciences, University of Birmingham, Birmingham, B15 2TT UK; 2 British Antarctic Survey, NERC, High Cross, Madingley Road, Cambridge, CB3 0ET UK; 3 University of Liverpool, Brownlow Hill, Liverpool, L69 7ZX England, UK; 4 School of Life Sciences, University of Warwick, Gibbet Hill Campus, Coventry, CV4 7AL UK; 5 Department of Zoology, University of Johannesburg, Auckland Park 2006, Johannesburg, South Africa; 6 Biodiversity of Antarctic and Sub-Antarctic Ecosystems (BASE), Santiago, Chile; 7 School of Geography, Earth and Environmental Sciences, University of Birmingham, Edgbaston, Birmingham, B15 2TT UK; 8 The Birmingham Institute of Forest Research, University of Birmingham, Edgbaston, Birmingham, B15 2TT UK; 9 Department of Entomology, University of Kentucky, Lexington, KY 40546 USA

**Keywords:** Archaea, Bacteria, Invertebrate, Nitrogen cycling, Nutrient release, Polar

## Abstract

*Eretmoptera murphyi* Schaeffer 1914 is a flightless chironomid midge endemic to South Georgia in the sub-Antarctic. In the 1960s it was accidentally introduced to Signy Island (in the more extreme maritime Antarctic), where it is now considered an invasive species. Detritivorous *E. murphyi* larvae can increase soil nitrogen levels by up to five times compared with similar uncolonized substrates, although the mechanisms involved remain unknown. This study conducted the first larval microbiome characterisation of *E. murphyi*, with the aim of identifying groups of microorganisms that may contribute to the elevated nutrient availability associated with this species. We also compare the *E. murphyi* microbiome with information available for other Antarctic invertebrates. Dominant archaea and bacteria included Crenarchaeota, Actinobacteriota, Chloroflexi, Proteobacteria and Planctomycetota, many of which have known roles in nutrient cycling. The microbiome of *E. murphyi* appears more diverse than that of other Antarctic invertebrates studied to date and includes phyla (Chloroflexi and Mycococcota) not previously reported from Signy Island soils or other Antarctic terrestrial invertebrate species. Further research is needed to establish which of these taxa represent true endosymbionts and to confirm their functional roles. The impact of non-native species microbiomes on nutrient cycling has important implications for polar terrestrial ecosystems, as significant changes in nutrient availability could impact native microarthropod and plant communities, as well as open new pathways for future non-native species establishment.

**Supplementary Information:**

The online version contains supplementary material available at 10.1007/s00248-026-02706-5.

## Introduction

The diversity and ecological success of insects is partly due to their associated microbiomes [1–3]. These communities of bacteria, archaea, fungi, protozoa and viruses influence many aspects of host biology [4], facilitated by long co-evolutionary relationships [5, 6]. Bacteria are the most abundant and diverse microorganisms present in the microbiome [4]. Mutualistic species can boost energy metabolism and play essential roles in nutrient acquisition [7], as well as in the host immune system [8], developmental processes [9] and fecundity [2]. Microbiome composition changes seasonally in some insects [10] and can contribute to winter/cold adaptation [11]. Studies of the relationships of archaea with their hosts are currently under-represented in the literature, primarily because this domain represents a smaller proportion of the microbiome compared to other groups [12]. Nonetheless, archaea can also play an important role in insect metabolism, such as in the fruit fly, *Anastrepha obliqua*, with communities changing across developmental stages and depending on diet [13]. Methanogenic archaea are important to digestion in detritus-feeding insects including beetles and termites [5]. Furthermore, as with gut bacteria, archaea can provide their insect hosts with additional nitrogen [5, 14], contributing to nitrogen cycling processes such as nitrogen fixation [15–17] and nitrogenous waste recycling [18].

**Fig. 1:**
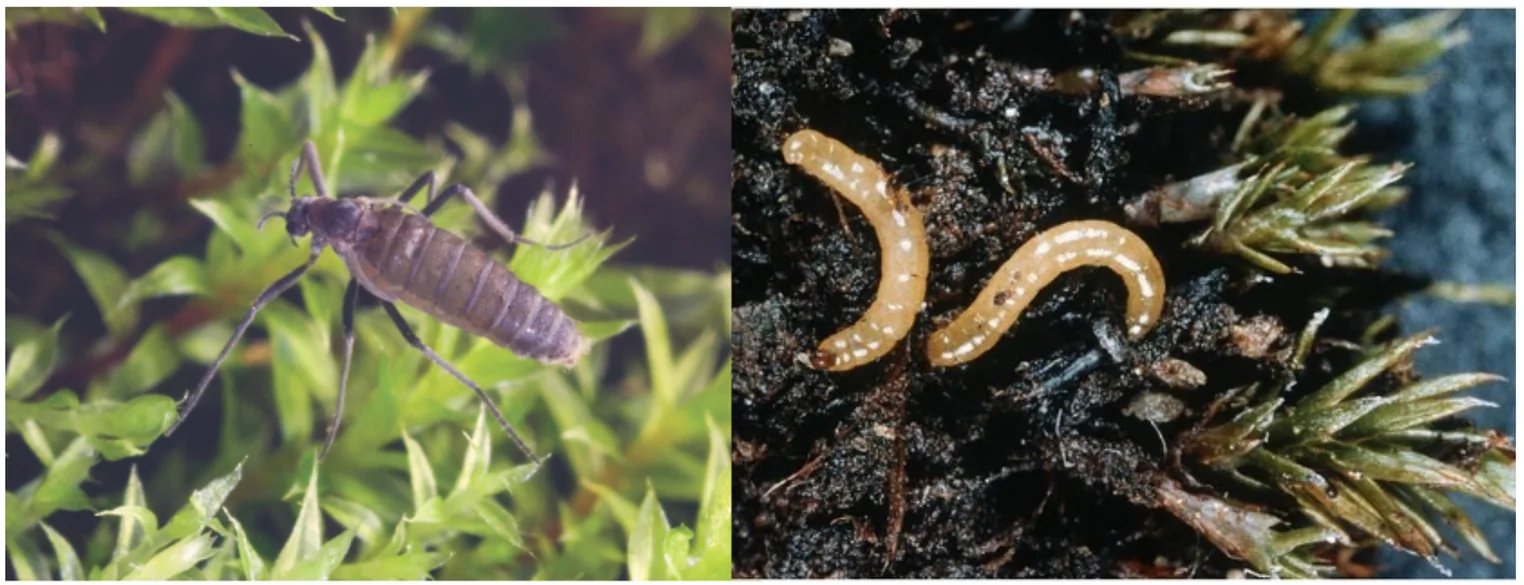
*Eretmoptera murphyi* Schaeffer 1914 [20] (Diptera: Chironomidae) adult (left) and larvae (right). Images: British Antarctic Survey

Investigating the microbiome of insects that inhabit typically nutrient-poor polar terrestrial ecosystems may provide unique insights into how these species establish and persist in these environments. Maistrenko et al. [19] characterised the gut microbiome of the Antarctic continent’s only endemic insect, *Belgica antarctica* Jacobs 1900 (Diptera: Chironomidae), noting a surprisingly limited overall diversity compared to temperate species. However, this study did not consider the nutrient cycling roles of any of the microorganisms identified. A closely related chironomid, *Eretmoptera murphyi* Schaeffer 1914 [20] (Diptera: Chironomidae; Fig. 1), is endemic to sub-Antarctic South Georgia and molecular phylogenetic analyses suggest that it is a sister species of *B. antarctica*, albeit with a multimillion-year evolutionary separation [21]. A recent study noted the absence of the bacterium *Wolbachia* in *E. murphyi* [22], as is the case in all Antarctic invertebrate microbiomes studied to date [23] but did not examine this species’ microbiome further.

*Eretmoptera murphyi* is now an established and invasive non-native species on Signy Island (South Orkney Islands, maritime Antarctic), where it was accidentally introduced most likely in the 1960s [24]. The fly’s larvae (Fig. 1) are detritivorous [25, 26] and their presence is associated with up to a fivefold increase in available soil nitrate compared with sites where only native invertebrates occur [27]. More recent data from our group indicates that concentrations of nitrate may be increased by an order of magnitude higher than these previous reports (Brayley et al., unpublished). Thus, while nutrient enrichment by soil fauna is a well-documented ecological process [28], native invertebrate detritivores (such as collembola and mites) are clearly not having the same impact on nutrient cycling as *E. murphyi*, particularly as the biomass of *E. murphyi* larvae can be greater than the entire native microarthropod and microinvertebrate community combined [26]. Given the usually nutrient-poor terrestrial Antarctic environment [29], this may have disproportionately large consequences on native microarthropod and plant communities and may also facilitate establishment of further non-native species [27, 30]. The spread of *E. murphyi* across Signy Island is associated with human footpaths [30] and recent observations have suggested that the insect appears to be locally increasing its range on the island (P. Convey, pers. obs.). It has been predicted that *E. murphyi* could increase litter turnover by up to 66.51 g dry mass m^− 2^ y^− 1^ [26], but it remains unclear whether the microbiome of *E. murphyi* could be facilitating an increase in soil nutrients.

The aims of this metagenomic study were to (1) conduct the first microbiome characterisation of *E. murphyi*, (2) suggest which groups of microorganisms present in the microbiome may contribute to the elevated nutrient availability associated with this species on Signy Island, and (3) compare the *E. murphyi* microbiome with information available for other Antarctic terrestrial invertebrates. In addition, given how little is known about Antarctic insect microbiomes, this study enhances understanding of the potential ecological roles of Antarctic invertebrates and contributes data to the region’s under-represented microbial reference databases.

## Methods

### Sample Collection

Larvae of *E. murphyi* were collected on Signy Island (South Orkney Islands, maritime Antarctic) during the austral summer in February 2023. They were obtained from the ‘Backslope’ (unofficial name; 60°42.6′S, 45°35.6′W [31]) adjacent to the British Antarctic Survey’s Signy Station on Berntsen Point, where the species is now patchily abundant [30]. The flora of the island, and specifically the study location, is dominated by cryptogams and extensive moss banks and carpets [27, 32], the soil is highly organic (mean soil organic carbon of 0.37 mg kg^− 1^ (Brayley et al. unpublished)), and has a mean pH of 4.54 [33, 34]. Larvae were kept in a container under field conditions on Signy Island before being rinsed and then stored in 96% ethanol at -80 °C during their return to the UK by ship. Samples were maintained at -80 °C at the University of Birmingham until analysis at the University of Warwick in April 2024. Seven larvae of the same size and developmental stage (L3 instar) were selected for metagenomic analysis to avoid introducing any differences associated with developmental stage [35, 36]. All larvae were checked to ensure absence of visible substrate on their cuticles before DNA extraction.

### Microbial DNA Extraction and Sequencing

Total DNA was extracted from each individual larva separately using the DNeasy PowerSoil Pro Kit (QIAGEN). Larvae were first manually fragmented with a sterilised pipette tip in solution C1 before the other extraction steps were carried out following the manufacturer’s protocol. Extracted DNA was quantified using a NanoDrop ND-2000 (Wilmington) and then used as a template for PCR amplification. Universal primers were used to target and amplify the bacterial V4 region of the 16 S rRNA, 515 F (5ʹ-GTGCCAGCMGCCGCGGTAA-3ʹ) and 806R (5ʹ- GGACTACHVGGGTWTCTAAT-3ʹ) [37]. Customised primers were also used to target and amplify the archaeal V4 region, 519 F (5ʹ-CAGYMGCCRCGGKAAHACC-3ʹ) and 806R (5ʹ-GGACTACNSGGGTMTCTAAT-3ʹ) [38]. Other PCR components were prepared using the NEBiolabs Q5 PCR kit following the manufacturer’s protocol. Two separate PCR reactions were carried out for each extraction, with 28 cycles for bacterial DNA and 32 cycles for archaeal (95 °C for 30 s, 53 °C for 30 s), with a final extension step at 72 °C for 30 s. The target region of ~ 300 bp was obtained by gel electrophoresis, followed by extraction and purification using the QIAquick Gel Extraction Kit (QIAGEN) following the manufacturer’s protocol. The concentration of recovered DNA was again quantified using a NanoDrop ND-2000 and the five samples with the highest concentrations of archaeal and bacterial DNA were selected for sequencing. Library preparation and sequencing were carried out commercially on an Illumina PE250 platform by Novogene (UK).

### Sequencing Analyses

The DADA2 pipeline (V1.32.0) [39] was used for quality trimming, error rate estimation, merging, chimera removal and amplicon sequence variant (ASV) feature table construction. A total of 333,809 bacterial and 355,215 archaeal reads were obtained. Primer sequences were removed from the 5’ region of forward and reverse reads (19 bp and 20 bp, respectively) and reads were truncated at the first instance of a quality score ≤ 10. Following dereplication, merging and chimera removal, 69.7–73.1% bacterial reads and 61.9–78.4% archaeal reads were retained for further analysis. Taxonomy was assigned to genus level using the SILVA 138.1 prokaryotic SSU taxonomic training data, formatted for DADA2 and Zenodo [40]. ASVs classified as chloroplast, mitochondria or archaea were removed from the bacterial sequence dataset, and those classified as chloroplast, mitochondria or bacteria were removed from the archaeal dataset. To remove sequences potentially arising from the host insect species, a local BLAST search was performed against the assembled genome of the closely related Antarctic chironomid *B. antarctica* [41] using rBLAST, as genomic sequence data are not available for *E. murphyi*. ASVs with > 90% sequence similarity across 90% of the query length were removed [42]. The bacterial and archaeal datasets were rarefied to depths of 42,723 and 31,182, respectively, using the rrarefy function from the R package vegan v 2.6–6.1, with default settings [43]. ASVs that represented ≥ 1% of the total abundance in either of the sequence datasets were classed as ‘dominant’ (for both bacteria and archaea). The most abundant bacterial ASVs at different taxonomic levels were defined as those with abundance in at least one individual (sample) of ≥ 10% for phylum, ≥ 6% for class, ≥ 25% for order, ≥ 10% for family and ≥ 5% for genus. The most abundant archaea were defined as those with abundance of ≥ 84% across all ranks. These thresholds were chosen based on the distribution of relative abundances across the individuals and to reflect the taxa that contributed considerably to the composition in at least one individual, avoiding including groups that were consistently found in low abundances or with sporadic representation.

### Statistical Analyses

Statistical analyses were carried out using RStudio, version 2024.09.1 + 394. Alpha-diversity indices (Chao1 richness, Shannon diversity) were calculated using the estimate_richness function in the *phyloseq* package [44] to quantify within-sample microbial richness and evenness. Beta-diversity measures were calculated using the phyloseq package with a Bray-Curtis distance metric [Supplementary Information (S1, S2)]. Differences in microbial community composition among the five larval samples were assessed using permutational multivariate analysis of variance (PERMANOVA; adonis2 function, vegan package), with ‘sample’ as the grouping variable. Associated statistics were calculated using PERMANOVA [45] with 999 permutations to obtain *p*-values, using the vegan function. The R² value was used to estimate the proportion of variation between larvae.

## Results

### Alpha Diversity

Across the five individual larvae analysed, the alpha diversity of the bacterial community was similar, with only minor variation in both richness (Chao1 index) and diversity (Shannon index) across individuals [Supplementary Information (S3)]. Alpha diversity of the archaeal communities showed slightly greater variation among individual *E. murphyi* larvae compared with the bacterial dataset. Chao1 richness values varied by approximately twofold across samples, whereas Shannon diversity indices showed only minor variation.

### Beta Diversity

Despite the differences in alpha diversity, there were no significant differences between the beta diversity (Bray-Curtis dissimilarity) of the bacterial (R^2^ = 0.37, *p* = 0.125) or archaeal samples (R^2^ = 0.18, *p* = 0.525).

### Bacterial ASV Diversity

**Fig. 2:**
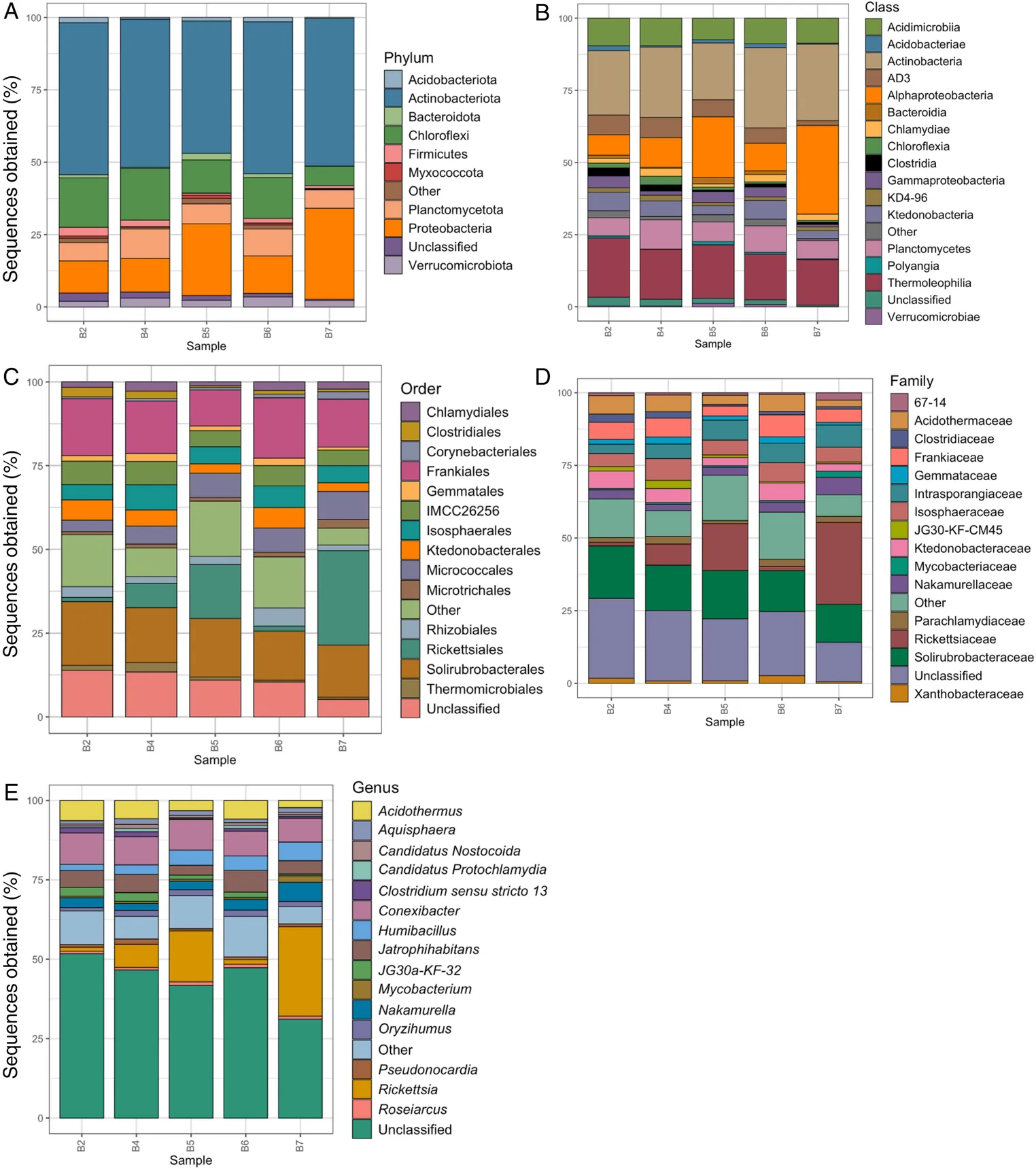
Stacked bar plots showing relative abundances (as percentage of sequences obtained) of the dominant (≥ 1%) bacterial phyla (**A**), classes (**B**), orders (**C**), families (**D**) and genera (**E**) detected in each individual *E. murphyi* larva (sample)

A total of 2013 bacterial ASVs were assigned. Of these, 1971 were classified to at least phylum level. A mean of 677 (range: 480–752) unique ASVs were detected in each individual larva. Bacteria therefore represented 97.4% of the total assigned microbiome. Representatives of 24 bacterial phyla were assigned. The most abundant phyla were Actinobacteriota, Chloroflexi, Planctomycetota and Proteobacteria (Fig. 2A). Of the 46 classes detected, the most abundant were Acidimicrobia, Actinobacteria, AD3, Alphaproteobacteria, Planctomycetes, Ktedonobacteria and Thermoleophilia (Fig. 2B). The most abundant of the 113 orders detected were Frankiales, Micrococcales, Rickettsiales and Solirubrobacterales (Fig. 2C). The most abundant families (148 detected) were Rickettsiaceae and Solirubrobacteraceae (Fig. 2D). Of the 200 genera assigned, the most abundant were *Acidothermus*, *Conexibacter*, *Humibacillus*, *Jatrophihabitans*, *Nakamurella* and *Rickettsia* (Fig. 2E). Only five ASVs were assigned to species, *Tomitella biformata*,* Methylocella palustris*, *Nakamurella panacisegeti*, *Faecalibacterium prausnitzii* and *Clostridium putrefaciens*.

### Archaeal ASV Diversity

A total of 47 ASVs were assigned using archaeal primers. Of these, 29 were assigned to archaea and classified to at least phylum level. Four ASVs were assigned to Eukaryota and the remaining 14 ASVs were not classified; these were not included in the subsequent analyses. A mean of 18 (range: 13–29) unique archaeal ASVs were obtained from the five individual larvae. Archaea comprised 2.6% of the total ASVs recovered. The phylum Crenarchaeota dominated the assigned sequences [Supplementary Information (S4)]. Representatives of the single class Nitrososphaeria were detected and all but one ASVs represented the order Nitrososphaerales (the exception was assigned to the Group 1.1c order), family Nitrososphaeraceae and genus *Candidatus Nitrocosmicus*. No ASVs were classified to species level.

## Discussion

Neither the overall microbial community composition (beta diversity) nor the relative abundance of archaeal and bacterial phyla differed significantly between the five individual larvae. This could be attributed to all samples being the same developmental life stage (L3 larvae) [35], as well as being sourced from the same habitat and soil type on Signy Island. These similarities suggest the presence of a small but consistent core microbiome across sampled individuals. It is not possible to confirm which of the archaea and bacteria assigned are true endosymbionts or commensal microorganisms of *E. murphyi*, because whole body DNA extractions may have detected microbial DNA on the external surface of larvae. However, below, we highlight if phyla have been reported previously in Signy Island soil samples and, where they have not, this provides additional support for an endosymbiont designation. Furthermore, some identified samples, such as the bacterial order Rickettsiales (phylum Proteobacteria, class Alphaproteobacteria), are known to be obligate intracellular organisms within eukaryotic cells [46]. In addition, one of the few bacterial ASVs assigned to species level, *Faecalibacterium prausnitzii*, is common in gut microbiomes [47].

**Fig. 3:**
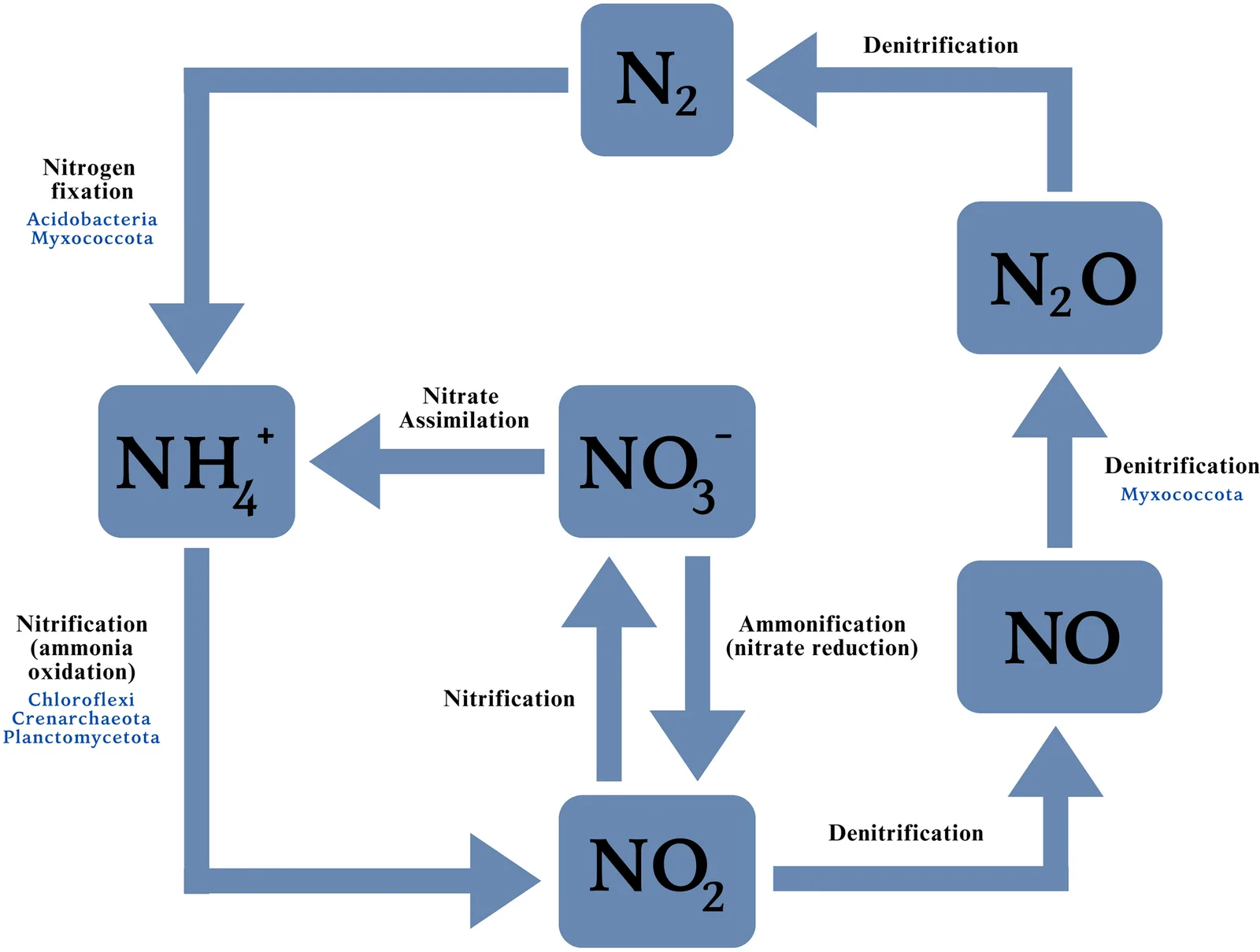
A simplified illustration of the soil nitrogen cycle, indicating candidate archaeal and bacterial phyla present in the microbiome of *E. murphyi* that may facilitate certain steps. A large proportion of microorganisms involved with nitrogen fixation and nitrification may contribute to the enhancement of nitrate concentrations in Signy Island soil. Re-drawn from Makhalanyane et al. [50]

Bartlett et al. [27] measured up to a 5 -fold increase in soil nitrogen, particularly nitrate, where *E. murphyi* larvae have established on Signy Island. More recently, we quantified nutrient concentrations in *E. murphyi*-infested soils and found that, in some locations, nitrate concentrations reached levels 24 to 39-fold higher than in uncolonised soils, (Brayley et al., unpublished). To account for this increase in total inorganic nitrogen, we sought to identify candidate microorganisms within the microbiome of *E. murphyi* that are involved in nitrogen cycling (Fig. 3). Specifically, those capable of nitrogen fixation, whereby atmospheric nitrogen is reduced to ammonia or ammonium ions. Also, groups of microorganisms were identified that are capable of ammonia oxidation, which is a limiting step during nitrification in the nitrogen cycle where ammonia is oxidised to nitrite [48]. Subsequent oxidation of nitrite to nitrate by other microorganisms is then required to release bioavailable nitrogen to plants and microarthropods [49]. This forms a foundation for future work to target these groups using functional or gene-level analyses.

### Bacteria

**Fig. 4:**
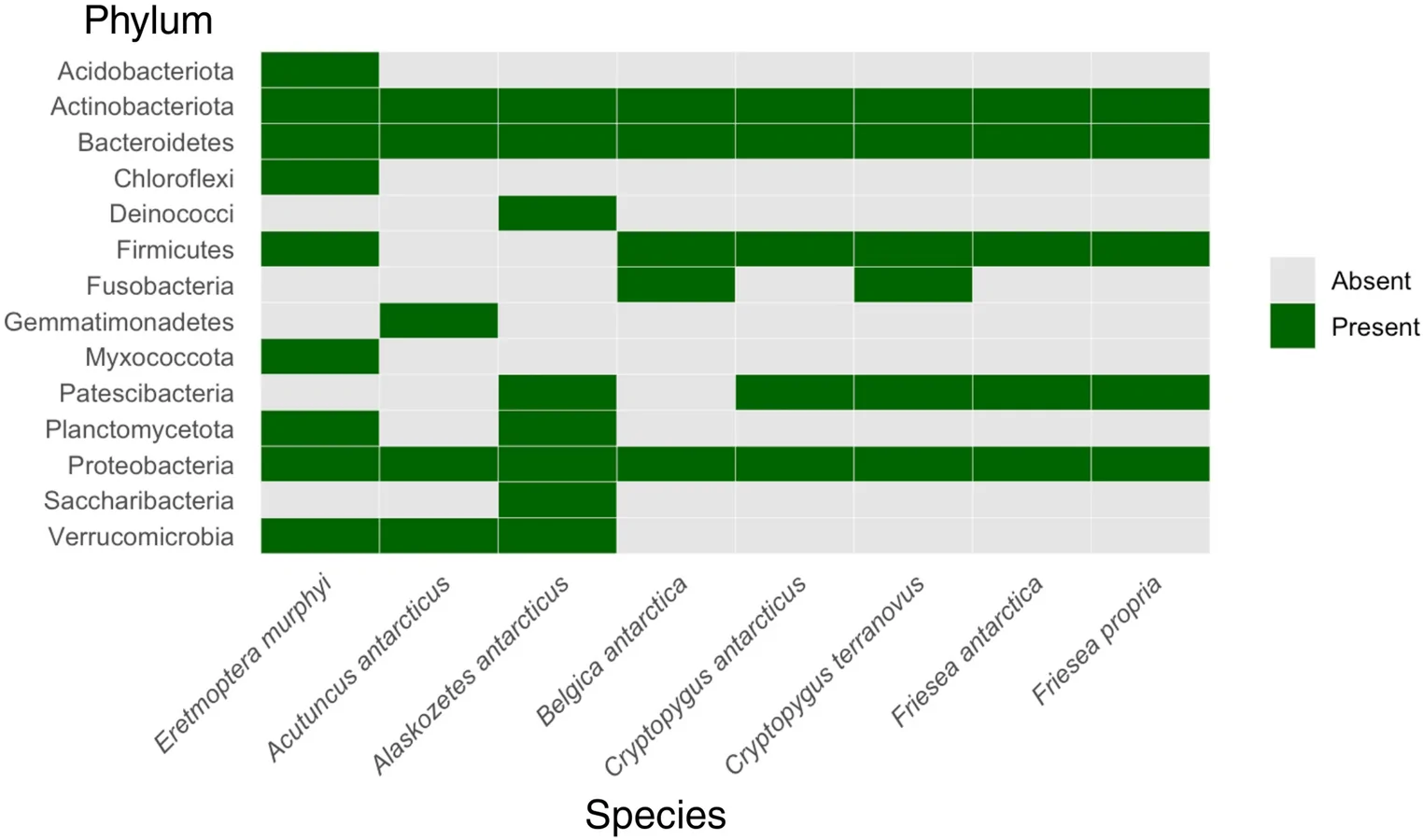
Presence/absence heat map comparing the most abundant bacterial phyla associated with *E. murphyi* and other studied Antarctic invertebrates. Collated from [19, 56–58]

The most abundant bacterial phyla in the *E. murphyi* microbiome were Actinobacteriota, Chloroflexi, Proteobacteria and Planctomycetota. One of these phyla, Chloroflexi, appears to be unique to *E. murphyi* when compared against other Antarctic invertebrate microbiomes (Fig. 4). Two less abundant phyla, Myxococcota and Acidibacteriia, also appear unique to *E. murphyi* (Fig. 4). Importantly, the collembolan, *Cryptopygus antarcticus*, and oribatid mite, *Alaskozetes antarcticus*, are dominant native terrestrial invertebrate species on Signy Island (and more widely in the Maritime Antarctic [51, 52] and are likely to have been present at many of the ‘control’ soil sample sites where Bartlett et al. [27] reported low nitrogen levels. Thus, any phyla absent from the microbiome of these two invertebrates, but present in *E. murphyi*, represent potential candidates to help explain increased nutrient availability associated with the non-native midge. Furthermore, Chong et al. [53] make no mention of Chloroflexi, or Myxococcota in their detailed study of spatial heterogeneity in the biodiversity of soil prokaryotes on Signy Island, which included collection sites where *E. murphyi* is known to occur. Acidibacteria have been reported in Signy Island soil [53, 54], while Yergeau et al. [55] report Chloroflexi in some of their soil samples from Signy Island. However, the latter study found them to be very low abundance, and the precise site of collection was not given. Thus, there is strongly suggestive evidence that many of the bacteria representing these phyla are more likely to have been *E. murphyi* endosymbionts rather than on its cuticle/contaminants from the soil.

Chloroflexi are functionally diverse, including chemoautotrophs, photoautotrophs and thermophiles [59]. While our understanding of Chloroflexi diversity, ecophysiology and metabolic functioning remains poor, there are several reported examples of nitrifying Chloroflexi [60–62]. Acidobacteria appear to be equipped with genes catalysing the metabolism of inorganic and organic sources of nitrogen, although clear experimental evidence of nitrogen fixation is lacking [63]. Investigations into the metabolic functions of Myxococcota suggest these bacteria are more commonly involved in denitrification [64]. However, a recent study of nitrogen cycling in coastal Antarctic systems identified several functional genes within metagenome-assembled genomes (MAGs) indicating that N_2_ fixation appears to be carried out by taxa within both Acidobacteriota and Myxococcota [65]. Planctomycetota was found at very low levels in the microbiome of *A. antarcticus* [56], is absent from *C. antarcticus* [57] and was not identified from soil at sites where *E. murphyi* is known to occur [53, 54]. This order has well-known roles in nitrogen cycling [66], particularly ammonia oxidation [65]. Notably, one of the dominant classes identified from *E. murphyi* samples was Planctomycetes (Fig. 2B), which is thought to have an important role in anammox pathways in Antarctic dry valley soil systems [67].

Actinobacteriota were abundant in the *E. murphyi* microbiome, but are also found in the microbiome of all other Antarctic invertebrates studied to date (Fig. 4). Representatives of this phylum are known to be highly tolerant of desiccation and other stresses [68] and can also play an important role in the carbon cycle, assisting with the decomposition of organic material and facilitating carbon sequestration in plants [69]. Within the abundant bacterial classes (Fig. 2B), Acidimicrobia (phylum Actinobacteria), are generally associated with low soil pH conditions (3.0–6.5) [70], consistent with the low pH of Signy Island soil. They play an important role in the carbon cycle, possessing genes associated with the breakdown of organic molecules such as carbohydrates (e.g., cellulose) and nitrogen-containing compounds (forming bacterial cellulose), and are significant decomposers within the soil community [63]. They may provide nutrition to *E. murphyi* by facilitating decomposition, as well as potentially conferring desiccation resistance through the formation of intestinal biofilms [71]. Studies of the genes associated with Ktendonobacteria (phylum Chloroflexi) have suggested that members of this class can carry out autotrophic carbon fixation [72, 73]. Such activity could reduce *E. murphyi*’s dependence on organic material as its primary carbon source [74]. Members of the family Solirubrobacteraceae (phylum Actinobacteriota, class Thermoleophilia) have been detected in desert environments exposed to high UV radiation, where they increase nitrogen availability and absorption [75], again potentially contributing to *E. murphyi* nutrient uptake.

### Archaea

The most abundant archaea, Crenarchaeota (also known as the Thaumarchaeota) [76, 77], are associated with sulphur-dependent thermophiles [78] and are generally found in high- or low-temperature environments [79]. Members of Crenarchaeota are known to perform ammonia oxidation [79–81] (Fig. 3). This process may be facilitated by the most abundant archaeal class identified in the microbiome here, Nitrosophaeria, whose members are typically found in nitrogen-limited and low pH soils, such as those occurring on Signy Island, as well as the McMurdo Dry Valleys [82]. Crenarchaeota have been reported in the guts of detritivorous insects, including termites (*Cubitermes orthognathus*) [83] and beetles (*Oryctes nasicornis* and *Amphimallon solstitiale*) [12]. This, combined with the particularly high abundance of Crenarchaeota found here, suggests that ammonia oxidation may contribute to the survival of *E. murphyi* larvae by allowing them to utilise the released nutrients for their growth. This may be a particularly important adaptation supporting the presence of the fly on Signy Island where, like much of Antarctica, nutrients (particularly nitrogen-containing compounds) are generally limited [84, 85]. Crenarchaeota have previously been reported from Signy Island (although not from soil), contributing 77% of the total sequences obtained from cryoconite holes [86]. Elsewhere in Antarctica, this phylum represented 80% of all archaeal sequences in soil from the McMurdo Dry Valleys [80, 87]. Such observations suggest that there may be a strong link between the archaeal microbiome of *E. murphyi* and that of the local environment.

### Comparison of *E. murphyi* microbiome with other Antarctic invertebrates

Maistrenko et al. [19] provide the only available data on the bacterial community associated with an insect endemic to the Antarctic continent, *B. antarctica.* This study combined data from both adults and larvae but only reported the presence of five bacterial phyla. In contrast, our *E. murphyi* (larvae only) dataset recovered 24 bacterial phyla (Fig. 2A), which is comparable to temperate chironomids such as *Chironomus ramosus* in India (22 phyla) [88] and *C. transvaalensis* in Israel (20 phyla) [35]. Four of the bacterial phyla identified in *B. antarctica* were shared with *E. murphyi* - Actinobacteria, Bacterioidetes, Firmicutes and Proteobacteria. The fifth, Fusobacteria, was not detected in the current study. Both insect species share representatives of two assigned genera, *Humibacillus* and *Pseudomonas*, although only two ASVs were assigned to these taxa here. Other genera assigned in *B. antarctica* but not detected in *E. murphyi* were *Arthrobacter*,* Cutibacterium*,* Porphyromonas*,* Lactococcus*,* Pelmonas*,* Janthinobacterium*,* Neisseria*,* Escherichia*,* Serratia* and *Yersinia*.

This comparison suggests that the bacterial communities associated with these two sister but biogeographically isolated insect species from the Antarctic region [21] are very different and unique. Maistrenko et al. utilised existing and archival whole-genome sequencing data to identify bacterial species associated with *B. antarctica*, which likely only gave a very limited characterisation as this is not a targeted approach. Further studies are also required to disentangle potential changes in each species microbiome across different developmental stages. As both species are detritivores [24, 89], it is likely that their gut microbial communities will reflect the substrate upon which they feed. Thus, some of the differences in microbial diversity found may be due to differences in the soil properties and associated microbial communities between the distinct geographical locations from which they originate. *B. antarctica* is endemic to the South Shetland Islands and western coastal regions of the Antarctic Peninsula, while *E. murphyi* is endemic to sub-Antarctic South Georgia with an introduced population on maritime Antarctic Signy Island [90, 91]. Interestingly, Yergeau et al. [55] reported several of the bacterial phyla unique to *E. murphyi*, Chloroflexi and Planctomycetota, in soil samples from South Georgia, so it is possible they were introduced to Signy Island with the non-native midge, given their absence from other Signy Island soil studies [53–55]. The highest densities of *B. antarctica* larvae are found in moss [92]; by contrast, *E. murphyi* on Signy Island favours dead organic matter and soil/peat substrata [30]. There may also be differences in the morphology of the gut [5] between the two species, although detailed gut description is currently only available for *B. antarctica* [89] and there are currently no studies that have examined the role of its microbiome in digestion or nutrient acquisition.

Leo et al. [57] employed metagenomic sequencing to investigate the bacterial microbiomes of four Antarctic springtail species, *C. antarcticus* (Collembola: Isotomidae) from the South Shetland Islands (maritime Antarctic), *Friesea antarctica* (Collembola: Neanuridae) from the South Shetland Islands and Lagoon Island (Marguerite Bay) and *C. terranovus* (Collembola: Isotomidae) and *F. propria* (Collembola: Neanuridae) from continental Antarctica (North Victoria Land). Only *C. antarcticus* occurs on Signy Island. Of the six most abundant phyla identified, all but Fusobacteria were shared with *E. murphyi*, and of the most abundant bacterial orders, only Corynebacteriales and Rhizobiales were shared with *E. murphyi*. As with *B. antarctica*, these springtail species had lower diversity bacterial microbiomes than *E. murphy*i.

Holmes et al. [56] characterised the bacterial microbiome of the Antarctic oribatid mite, *Alaskozetes antarcticus* (Oribatida: Trhypochthoniidae), from Cormorant Island, south of Anvers Island off the western Antarctic Peninsula. This species also dominates the invertebrate biodiversity in several habitats on Signy Island [93]. The dominant bacterial phyla detected in the mites (males, females and tritonymphs) were Actinobacteriota, Bacteroidetes, Deinococci, Patescibacteria, Planctomycetota, Proteobacteria, Saccharibacteria and Verrucomicrobiota, which are all shared with *E. murphyi* apart from Deinococci, Patescibacteria and Saccharibacteria (Fig. 4). Only two abundant genera are shared between the species, *Mycobacterium* and *Nakamurella.*

Finally, Vecchi et al. [58] assessed the bacterial microbiome of the Antarctic tardigrade, *Acutuncus antarcticus* (Parachela: Hypsibiidae), from Victoria Land (continental Antarctica). This, again, suggested many similarities with *E. murphyi* in terms of the shared phyla, Actinobacteria, Bacteroidetes, Proteobacteria and Verrucomicrobia, with only Gemmatimonadetes not found in our study (Fig. 4). There are no shared genera between the tardigrade and *E. murphyi.* Overall, the relatively diverse microbiome of *E. murphyi* differs from other Antarctic invertebrates examined to date, although the possible influence of methodological differences cannot be discounted. Finally, not all previous studies have characterised microorganisms to genus level, and those that have all utilise different threshold levels and pipelines, so in-depth comparisons remain difficult.

### Future Work

To test hypotheses on whether *E. murphyi*’s microbiome has facilitated its success as an invader on Signy Island, future research must address which archaea and bacteria are true endosymbionts of *E. murphyi*. This could be achieved by using bleach to remove external microorganisms [94] or dissecting the digestive tract and isolating the associated microorganisms [95]. Analysis of microbial community differences between larval stages and the non-feeding adults would also contribute. Access to fresh material from the species’ native South Georgia, where the fly is increasingly hard to find within areas recently colonised by non-native and aggressive predatory beetles [96, 97], would also allow assessment of the extent to which the fly’s microbiome has altered in the c. 60 years since its introduction to Signy Island. Although amplicon sequencing is a powerful tool, it should be noted that this methodology does not confirm the activity of microorganisms, nor does it differentiate between expressed and non-expressed genes [98]. To resolve this, a combination of ‘omics’ technologies could be applied, including metatranscriptomics, to assess the activity of the microbiome community [75], their associated functions in insect nutrition [99] and, potentially, the wider environment.

## Conclusions

We provide the first characterisation of the archaeal and bacterial microbiomes associated with *E. murphyi*, and invasive non-native insect on Signy Island, maritime Antarctic. The most abundant archaeal and bacterial phyla were Crenarchaeota, Actinobacteriota, Chloroflexi, Proteobacteria and Planctomycetota. Chloroflexi appears to be unique to *E. murphyi*, compared to the microbiomes of native invertebrates on the island, and has not been found in soil studies at *E. murphyi* sites. Planctomycetota, also appears to be absent from soil at *E. murphyi* sites and has only been identified in one other Antarctic invertebrate. Two less abundant phyla, Acidobacteriota and Myxococcota are unique to the *E. murphyi* microbiome and Myxococcota has not been previously reported from Signy Island soils. All have known roles in nitrogen cycling and represent candidates to explain the greatly increased soil nitrogen levels associated with midge larvae. Representatives of these phyla may allow *E. murphyi* to access additional energy sources in the nutrient-depleted soils of Signy Island which, in turn, could release key nutrients into the peaty soil. *Eretmoptera murphyi* appears to have a more diverse microbiome (at the phylum level) than several native Antarctic invertebrates studied to date, but differences in methodologies across studies currently limit robust comparisons. Further research is required to confirm which organisms are true endosymbionts/commensals and directly assess their functional roles. Finally, comparison of the Signy Island *E. murphyi* microbiome with that of the original source population from South Georgia would enable the first confirmation of the combined transfer of microbiota in an introduction event to Antarctica.

## Supplementary Information

Below is the link to the electronic supplementary material.

Supplementary Material 1

Supplementary Material 2

Supplementary Material 3

## Data Availability

The ASVs generated during the current study and their taxonomic assignment are available in the Supplementary Information. The accession numbers are available in the SRA database under the following BioProject: PRJNA1370479.
