# Supplementary material for: The Microbiome of an Invasive Antarctic insect, Eretmoptera Murphyi (Diptera: Chironomidae), and its Potential Role in Nutrient Cycling
Source: Microb Ecol. 2026 Feb 28;89:66. doi: 10.1007/s00248-026-02706-5 (PMC12966229; doi:10.1007/s00248-026-02706-5)
Supplement: Supplementary file 1 [file 248_2026_2706_MOESM3_ESM.docx]

**Supplementary Information**

Article title: The microbiome of an invasive Antarctic insect, *Eretmoptera murphyi* (Diptera: Chironomidae), and its potential role in nutrient cycling

Journal name: Microbial Ecology (Insect Microbiome Collection)

Authors

Octavia D. M. Brayley^1,2^ ([oxb233@student.bham.ac.uk](mailto:oxb233@student.bham.ac.uk); <https://orcid.org/0000-0003-1826-543X>), Kirsty McCready^1^ (<https://orcid.org/0000-0002-1175-5973>), Shengwei Liu^3^ (<https://orcid.org/0000-0001-6418-2884>), Peter Convey^1,2,4,5^ (<https://orcid.org/0000-0001-8497-9903>), Yin Chen^1^ (<https://orcid.org/0000-0002-0367-4276>), Sami Ullah^6,7^

(<https://orcid.org/0000-0003-0963-7457>), Nicholas Teets^8^

(<https://orcid.org/0000-0002-9153-8847>), Scott A.L. Hayward^1,7^ (<https://orcid.org/0000-0002-1899-6630>)

^1^School of Biosciences, University of Birmingham, Birmingham, B15 2TT, United Kingdom

^2^British Antarctic Survey, NERC, High Cross, Madingley Road, Cambridge, CB3 0ET, United Kingdom

^3^School of Life Sciences, University of Warwick, Gibbet Hill Campus, Coventry, CV4 7AL, United Kingdom

^4^Department of Zoology, University of Johannesburg, Auckland Park 2006, South Africa

^5^Biodiversity of Antarctic and Sub-Antarctic Ecosystems (BASE), Santiago, Chile

^6^School of Geography, Earth and Environmental Sciences, University of Birmingham, Edgbaston, Birmingham, B15 2TT, United Kingdom

^7^The Birmingham Institute of Forest Research, University of Birmingham, Edgbaston, Birmingham, B15 2TT, United Kingdom

^8^Department of Entomology, University of Kentucky, Lexington, Kentucky, 40546, United States of America

**Fig. S1** Principal coordinates analysis (PCoA) of microbial community composition based on Bray–Curtis dissimilarity. PCoA plot showing variation in microbial communities across bacterial samples (n = 5). Each point represents one sample, coloured according to sample ID (2, 4, 5, 6, 7). Axis labels indicate the proportion of total variation explained by the first two principal coordinates (PCo1 = 51.2%; PCo2 = 20.1%).

**Fig. S2** Principal coordinates analysis (PCoA) of microbial community composition based on Bray–Curtis dissimilarity. PCoA plot showing variation in microbial communities across archaeal samples (n = 5). Each point represents one sample, coloured according to sample ID (2, 4, 5, 6, 7). Axis labels indicate the proportion of total variation explained by the first two principal coordinates (PCo1 = 51.2%; PCo2 = 20.1%).

**Fig. S3** Alpha diversity of bacterial (above) and archaeal (below) communities associated with five *Eretmoptera murphyi* larvae (samples). Diversity was assessed using the Chao1 (richness) and Shannon (richness and evenness) indices. Each point represents a single larva.

**Fig. S4** Archaeal 16S rRNA gene ASVs classified by phylum obtained from five *E. murphyi* larvae. Each point represents the relative proportion (%) of 16S rRNA gene copies assigned to either Crenarchaeota or an unclassified taxon. Horizontal black lines represent the boxplot median; in these data Q1 and Q3 equal the median, so each box collapses to a single line.
